# A Presumptive Case of Human Rabies: A Rare Survived Case in Rural Ghana

**DOI:** 10.3389/fpubh.2016.00256

**Published:** 2016-11-11

**Authors:** Paschal Awingura Apanga, John Koku Awoonor-Williams, Michael Acheampong, Matthew Ayamba Adam

**Affiliations:** ^1^Ghana Health Service, Talensi District Hospital, Tongo, Ghana; ^2^Policy, Planning, Monitoring and Evaluation Division, Ghana Health Service, Accra, Ghana; ^3^Ghana Health Service, Regional Hospital, Bolgatanga, Ghana

**Keywords:** rabies, human rabies, dog bite, vaccination, surveillance

## Abstract

Rabies remains endemic in Ghana and continues to pose a major public health threat to humans and animals with a nearly 100% case fatality rate in humans. We report of a presumptive case of human rabies whose survival represents a rare occurrence in rural Ghana and worldwide. Lessons from this case study provide a critically needed focus in helping improve rabies surveillance and case management in Ghana. We report of the survival of a 36-year-old man who developed clinical rabies after he was bitten by his dog, while restraining the dog with a chain. Prior to this, he did not observe any abnormal or rabid behavior in the dog. Following the bite, he did not immediately resort to hospital treatment, but rather to traditional application of herbs to the laceration he sustained after the bite. The reason given for not seeking immediate hospital treatment was that the dog was not rabid and lack of funds to seek hospital care. However, after 10 days he began to show symptoms of confusion, hydrophobia, and photophobia, consistent with rabies virus infection, and was subsequently rushed to the hospital by relatives. At the hospital, he was administered human immune tetanus immunoglobulin, diazepam, ceftriaxone, paracetamol, and intravenous fluids. No rabies vaccine was administered. Six days after commencing treatment, the patient became well, showed neither signs of confusional state, hydrophobia, nor photophobia. He was discharged home after 13 days of commencing treatment. This study provides insight on a presumptive case of human rabies that survived despite non-administration of rabies vaccine after exposure. It also exposes the weaknesses in the health and veterinary systems in rural Ghana regarding rabies surveillance and case management.

## Introduction

Rabies is an infectious viral disease with an almost 100% case fatality rate in humans (1, 2). Rabies is caused by lyssavirus which causes an acute viral encephalomyelitis (3). Globally, about 15.69 million people are exposed to the rabies virus annually with an estimated 59,000 human deaths (3, 4). Rabies remains a public health threat particularly in developing countries like Africa and Asia, which accounts for more than 95% of all human rabies cases (5, 6).

Rabies is a neglected tropical disease of the poor and vulnerable populations with deaths due to rabies often not reported (6). High incidence of rabies morbidity and mortality among this population has been reported as human vaccines and immunoglobulin are not readily available or accessible. About 99% of rabies transmission to humans is by the bite or scratch of infected domestic dogs, although transmission by wild animals, such as bats, foxes, and wolves, has also been reported (3, 7, 8). The incubation period for rabies usually varies from 1 to 3 months, but may also vary from less than a week to greater than a year depending on the age, location of bite, and the rabies viral load on the human (6, 9).

Two forms of clinical presentation of human rabies, furious and paralytic rabies, have been observed. Furious rabies accounts for about 67% of rabies cases and is characterized by hyperactivity, excited behavior, hydrophobia, aerophobia, delirium, reduced consciousness, and occasional seizures. Paralytic rabies account for approximately 33% of human rabies cases and causes paralysis of limbs and respiratory muscles (3, 6). When human rabies is not treated, death ensues within 5–7 days after the onset of symptoms. Treatment of rabies may prolong survival span of patients by 133 days (2). Deaths in human rabies cases are usually due to cardiopulmonary arrest (10).

Currently, there are no available tests that can confirm rabies virus infection in humans before the onset of clinical disease. Clinical diagnosis of rabies is often made when rabies-specific signs, such as hydrophobia or aerophobia, are present (6). Human rabies can be confirmed during clinical disease stage and post mortem by detecting viral antigens, whole virus, or nucleic acids in infected tissues (brain, skin, urine, or saliva) using various diagnostic techniques. Although laboratory diagnosis of rabies infection in humans is difficult after exposure to the virus, post-exposure prophylaxis has helped reduce the risk of acquiring rabies infection in an exposed individual (11, 12). Post-exposure prophylaxis consist of flushing and washing the site of the bite or scratch with soap and water, detergent, and an antiseptic as well as administration of rabies immunoglobulin (RIG).

In Ghana, rabies is endemic, and cases of human rabies are under reported as in other developing countries (13). Veterinary services in Ghana are often limited in the diagnosis of rabies, as Sellers’ stain and fluorescent antibody test, commonly used techniques in diagnosing rabies, are mostly unavailable. The clinical diagnosis of human rabies of a person bitten by a dog is usually confirmed by the veterinary services if a dog tests positive for rabies (14, 15). Preventive and control measures in Ghana to reduce the incidence of human rabies have been targeted at improving the vaccination of dogs against rabies, stray dog removal, and providing pre-exposure/post-exposure vaccinations of humans; however, these measures have been irregular and not sustained (14, 15).

The Bolgatanga regional hospital in the Upper East Region (UER) of Ghana has not recorded any survived presumptive cases of human rabies from data available since 2013 (16). However, we recently encountered a presumptive case of human rabies who survived. Very little evidence has been documented about human rabies in Ghana, and this case provides useful case study for clinicians and veterinary practitioners working in rural Ghana.

## Case Presentation

On the 2nd of February, 2016, a 36-year-old man from Dubila, a rural community in the Bolgatanga Municipality of the UER, was bitten by his 1-year-old unvaccinated dog when he tried to chain it and sell to a buyer from a neighboring community. Although he succeeded in selling the dog, he sustained a 3 cm deep laceration on the posterior part of proximal third of the right leg. He resorted to treatment from a traditional herbalist who gave him the ground bark of a mahogany tree (*Swietenia macrophylla*) to apply to the wound he sustained. According to him, his dog never exhibited any abnormal or rabid behavior, and he did not have money to seek care at the local hospital. He had no past history of pre-/post-exposure prophylaxis vaccination. A week later he started experiencing prodromal symptoms in the form of fever, headache, and general malaise. However, he was unperturbed as he continued his herbal medication with the hope that he would recover fully.

On the 12th of February, he was rushed to the Bolgatanga regional hospital by his relatives with signs of agitation, confusion, photophobia, and hydrophobia which are classical symptoms of rabies virus infection. A presumptive diagnosis of human rabies was made on the first day of admission. He was admitted in an isolation ward and, on examination, the cardiovascular and gastrointestinal systems were observed to be normal. Neurological examination revealed normal cranial nerves and pupils equal and reacted briskly to light. Neck rigidity was absent with muscle spasms in all limbs. His mental state could not be fully assessed due to his state of agitation. His blood pressure measured was 120/70 mmHg, temperature 37.9°C, while his pulse and respiratory rates were 70 beats per minute and 14 breaths per minute.

Although the hospital lacked the capacity to perform basic tests that could isolate the viral antigens to help support the diagnosis of rabies virus in the patient, doctors informed the veterinary services and with the support of the relatives of the patient they located the buyer of the dog that bit him. The dog was still alive and then put to death. A confirmation of rabies infection was made by direct fluorescent antibody test on the dog’s brain tissue. Blood glucose, serum electrolytes, liver function, and renal function test of the patient were normal. However, his full blood count showed high white blood cell count, but a lymphocytosis with normal neutrophil count. Lumbar puncture was conducted and cerebrospinal fluid was normal. The patients’ relatives were counseled on the diagnosis and prognosis.

The patient’s wound was cleaned thoroughly and dressed on a daily basis. He was administered intramuscular injection of 250 IU of human immune tetanus immunoglobulin and placed on broad spectrum antibiotic (ceftriaxone) as well as intravenous fluids. On the 13th of February, the patient became aggressive and was still in a confused state with hydrophobia. He was administered intravenous diazepam. Ketamine was not readily available for use. However, after administration of the diazepam, the patient responded very well to treatment. On the 18th of February, the patient’s condition improved drastically. He became oriented in time, place, and person, with no photophobia and hydrophobia. His only complaint was intermittent headache with restlessness. He was administered paracetamol for the headache. Treatment continued until the 25th of February when he was discharged home as his condition became much better and he complained of only mild headache. He was reviewed after a month, 25th of March, 2016 and was declared clinically fit. Subsequent monthly follow up reviews revealed he was clinically fit without residual neurological symptoms with the last review on 11th of July, 2016.

## Discussion

The findings in this case provide evidence for the need to strengthen health care and veterinary services in Ghana. The application of herbs from a traditional herbalist for the laceration sustained from the dog bite has exposed the lack of knowledge and understanding in seeking care and treatment for rabies infection. Interestingly, even though the patient revealed that financial constraints was one of the reasons for not seeking care at a health facility, he was able to pay the bills when he was treated at the hospital. We observed that the patient did not vaccinate his 1-year-old dog and was also unaware of post-exposure prophylaxis vaccine against the rabies virus. This situation emphasizes the need for policy makers to implement interventions that are targeted at public health education and awareness creation on the importance of seeking health care at health-care facilities, rather than resorting to traditional herbal treatment. The veterinary services need to be well resourced to be able to carry out household vaccination of dogs and continuously create awareness on the importance of vaccination and the need to seek care immediately at a health facility when one is bitten by a dog. Government should also ensure that RIG is freely available for clients who may require it in health facilities in the country.

The application of herbs (bark of mahogany) to the wound sustained after the dog bite might have contributed to the recovery of the patient. Even though there is no established evidence on the role of the herbs use in the treatment of rabies virus infection, the herbs might have had some therapeutic effects. Studies have shown that, besides the use of mahogany as timber, it has been found to have benefits in phytomedicine because of the variety of biological activities it provides, including treatment of viral infections (17–19). In Ghana, mahogany is used for timber and herbs for the treatment of malaria, anemia, headaches, diarrhea, and skin infections (20–22).

This report found that the regional hospital, which is the main referral facility in the region, lacks the requisite equipment to carry out tests [computerized tomography (CT-scan), magnetic resonance imaging (MRI), polymerase chain reaction (PCR), fluorescent antibody test] to support a clinical diagnosis of human rabies. If the hospital had available a CT-scan or MRI, it could have been used to rule out an intracranial space occupying lesion making our presumptive diagnosis of rabies more likely. The regional hospital also lacked the capacity to determine the patient’s immune status to rabies virus infection. This situation needs to be addressed and health facilities in the region should be well equipped in the diagnosis and management of human rabies. Although the hospital had no capacity to confirm the case of human rabies, samples of CSF should have been taken from the patient, stored, and sent to the veterinary services in Tamale, the capital of the Northern region to confirm whether it was a case human of rabies.

The action of the veterinary services working upon information from the health staff in tracing and locating the buyer and confirming the diagnosis of rabies in the dog is commendable and provides an important perspective to the One Health Approach. Working together with health authorities, awareness creation must be intensified in the local population, who buy dogs, to request for evidence of vaccination against rabies before purchasing as they might risk exposure to rabies virus. The absence of regular vaccination for dogs has been highlighted.

It was observed that even after confirmation of rabies in the dog, neither the relatives of the patient, the buyer of the dog, his family, nor dogs in this community were vaccinated. This raises concern of spread of infection. We recommend that contact tracing should be done to ensure that persons who have been in contact with a rabid dog receive vaccination as well as animals that might have been infected by the rabid dog.

## Conclusion

This case report has provided valuable lessons for rabies surveillance and case management in northern Ghana. The lack of regular vaccination of dogs in communities poses high risk of infection of dogs, and its associated dangers to the populations and public health. The case highlights the use of herbs (bark of mahogany) in the treatment of rabies although there is no established evidence for the role of the herbs used to treat human rabies virus infections. It has also provided insight on how a presumptive case of human rabies survived despite not receiving rabies vaccination after exposure. There is a strong need for future research on the efficacy of the herbs that were used as it might have some therapeutic effects on rabies case management.

## Ethical Approval

Ethical Approval was given by the Upper East Regional Health Directorate. Approval was then provided by the Bolgatanga Regional Hospital, which is a health facility that operates under the Upper East Regional Health Directorate. Consent and approval was also given by the participant for the study and publication. Participant was well informed about the study and right to withdraw from the study even after participation.

## Author Contributions

PA conceived the study. PA, MAA, MA, and JA-W designed the study and collected the data. PA and MA analyzed the data and wrote the first draft. JA-W critically revised the draft for important intellectual content. All the authors read and approved the final manuscript.

## Conflict of Interest Statement

The authors declare that the research was conducted in the absence of any commercial or financial relationships that could be construed as a potential conflict of interest.

## Abbreviations

CT, computerized tomography; MRI, magnetic resonance imaging; PCR, polymerase chain reaction; RIG, rabies immunoglobulin; UER, Upper East Region.
